# Rollator usability from a nursing science perspective: A parallel qualitative content analysis of customer reviews on Amazon

**DOI:** 10.1016/j.ijnsa.2025.100422

**Published:** 2025-09-11

**Authors:** Marcel Schmucker, Andreas Küpper, Laura Hahn, Cornelia Mahler, Astrid Elsbernd

**Affiliations:** a Institute for Health Care and Nursing Sciences, Esslingen University of Applied Sciences, Germany; b Department of Nursing Science, Institute for Health Sciences, University Hospital Tübingen, Germany

**Keywords:** Amazon, Ambulation aids, Consumer satisfaction, Online product reviews, Rollators, Usability Study, Walkers

## Abstract

**Background:**

Care dependency and mobility restrictions often go hand in hand, increasing the risk of falls. Rollators are essential assistive devices that support individuals’ mobility and functioning, with globally varying usage rates. While overall user satisfaction is rated high, usability challenges persist. Beyond advocating for user needs, nursing science should also address the role of rollators in enabling individuals to remain at home despite care needs, ensuring safety, and shaping informal and formal care settings.

**Purpose:**

This study examined usability aspects with a strong user-centred emphasis on subjective rollator satisfaction, using Amazon rollator reviews as data source. The aim was to validate previously found aspects and to explore unknown elements of human-rollator interaction.

**Methods:**

A total 1.026 rollator reviews from three price categories (200 €) were analysed. A Qualitative Content Analysis was employed, combining deductive and inductive coding methods. Deductive analysis was conducted based on the Quebec User Evaluation of Satisfaction with assistive Technology (QUEST 2.0). Inductive analysis was based on the Grounded Theory Method to identify themes that were not fully considered in the deductive coding.

**Results:**

A total of 2243 deductive codes were assigned, with Ease of Use (489) and Comfort (458) being the most frequently coded categories. The inductive analysis revealed that users' expectations differ depending on the objectives of the primary and secondary users, who often make the purchasing decisions. Aesthetic appeal influenced rollator acceptance and reducing stigma. Usability has been shown to evolve over time, with experience, adaptation, and wear affecting long-term satisfaction and maintenance needs.

**Conclusions:**

This study highlights the complexity of rollator usability, shaped by material and non-materialistic user needs. Amazon reviews offer valuable insights, including secondary user perspectives. Nurses can play a key role in training and advising on rollator, contributing to better provision. As rollators shape care situations, it is essential for nursing science to address assistive technology to improve usability, safety, and overall quality of care.

**Study Registration:**

Not registered.

**What is already known**

• Rollators help reduce care dependency from mobility limitations.
• Rollators are prone to incorrect handling, affecting users’ health and social interactions.

Alt-text: Unlabelled box

**What this paper adds**

• Nurses could play a key role in rollator provision by filling gaps in follow-up care, teaching and training.
• This is the first qualitative study on rollator usability based on Amazon reviews.

Alt-text: Unlabelled box

## 1 Background

Rollators are listed on the *Priority Assistive Products List* as essential devices that are highly needed and a necessity to maintain or improve individuals’ mobility and functioning (WHO, 2016). Rollators are assistive devices designed to provide users with stability and safety while standing and walking. They are often equipped with a seat and a basket/bag, offering users the ability to rest when needed and transporting of items (ISO, 2021). Globally, the *Measuring Access to Assistive Technology* report highlights a low median prevalence of rollator need (0.1 %) and an access rate of 12.3 % (WHO, 2022). In contrast, rollator usage in Northern and Central Europe appears significantly higher, as evidenced by Sweden reporting 17,761 annual rollator prescriptions per million citizens (Socialstyrelsen in WHO, 2022) and Germany recording 425,227 sales in 2012 (MTD-Verlag, 2012).

While overall satisfaction with rollator use has been reported as high (Schmucker et al., 2024), human-rollator interactions are prone to malfunctions, including accidents or misuse due to a lack of usage knowledge (van Riel et al., 2014). Misuse or non-use of technology is often linked to poor usability, resulting from bad design (Norman, 2013), where devices fail to adequately address its intended purpose. To prevent these issues, medical devices such as rollators must meet usability requirements for efficiency, effectiveness, and satisfaction (Regulation (EU) 2017/745). However, current knowledge on rollator usability is mostly limited to quantitative parameters of efficiency and effectiveness, as shown in the rollator regulations of the International Organization for Standardization (2021), with qualitative aspects like user satisfaction remaining underexplored (Schmucker et al., 2024). Addressing these gaps, WHO recommends that assistive devices should be safe, effective, and affordable, while also emphasising the empowerment of users and their families to perform basic repairs, maintenance, adaptions and to actively involve them in design processes. Furthermore, WHO highlights the need to destigmatise assistive technology through improve product design (WHO, 2022). Whether assistive devices like rollators are well-designed and effectively implemented should concern nursing science, not only in terms of advocacy for users (ICN, 2021) but also in relation to their effects on enabling individuals to remain at home despite care needs, ensuring user safety (DNQP, 2020), and shaping the working conditions of both formal and informal caregivers (Manz, 2015). Care dependency is often accompanied by mobility restrictions and an increased risk of falls (Musich et al., 2018), directly impacting autonomy of individuals in performing their daily Activities of Living (Roper et al., 2000). Primary users, relatives, and nurses, as secondary users, evaluate satisfaction with assistive devices based on categories such as safety, ease of use, weight, and repairs, as reflected in the QUEST 2.0 categories (Demers et al., 2002). Choosing the right assistive devices is crucial here, as requirements can vary individually. Assistive device consultation is important to avoid negative impacts on individual users, their relatives, and to ensure successful care (DNQP, 2020).

For this reason, the focus of this study was on examining usability from a nursing science perspective, with a strong user-centred emphasis on subjective rollator satisfaction. We placed less emphasis on aspects of user experience or the explanation of rollator accessibility through technology acceptance models, although these topics were partially addressed within the broader context of usability, where these aspects overlap. Amazon rollator reviews served as data basis for this study, offering convenient access to user evaluations. These reviews provided an opportunity to validate previously identified aspects from our literature review findings (Schmucker et al., 2024) and to explore unknown elements of human-rollator interaction, thereby revealing additional aspects of usability.

## 2 Methods

### 2.1 Qualitative approach

This study is based on a constructionist perspective and uses a pragmatistic research approach, applying a Qualitative Content Analysis (Mayring, 2022). We do not see usability as an objective or fixed property, but as something that emerges through use and through ongoing interactions between people and technology (Janda, 2019). The theoretical basis is Actor-Network Theory, which views both people and objects as active participants in interaction (Latour, 2019). In this view, both users and rollators work together to enable daily activities like walking, sitting, or carrying items. Additionally, we apply the concept of affordances, which describes how physical objects, like rollators, offer possibilities for use (Gibson 1986 in Schulz-Schaeffer, 2021). This is further supported by the belief that good rollator design allows users to easily recognise and intuitively use the offered product affordances (Norman, 2013). From a pragmatistic perspective, only themes with practical relevance for rollator users are pursued, without allowing themes to be imposed or neglected by committing to a deductive or inductive methodological approach. For this reason, we used a Qualitative Content Analysis in the form of a parallel procedure, in which we conducted a rigorous rule-led analysis via deductive coding and an open interpretative analysis based on inductive coding. The rationale for this lies in the already broad theoretical and normative knowledge about usability and its components (Janda, 2019, International Organization for Standardization 2018), such as satisfaction, which can be well described using theory frameworks of established measurement tools such as QUEST 2.0, where satisfaction with assistive technology is assessed in eight device and four service dimensions (Demers et al., 2002). On the other hand, certain aspects of rollator-human interaction, and thus the characteristics of individual usability, remain theoretically largely unclear. Because of this, an exploratory interpretative approach seems appropriate.

### 2.2 Researcher characteristics and reflexivity

Our research group consists of nursing scientists with varying levels of experience in qualitative methods and extensive expertise in clinical, long-term, and outpatient care, as well as in the development of nursing and health technologies.

### 2.3 Context

Amazon product reviews consist of a quantitative rating (stars ranging from 1 to 5, from very poor to excellent) and a qualitative review section where users can provide free-text evaluations. The qualitive review can vary in length and format and users have the option to upload images and videos. Product reviews have a significant impact on purchasing decisions and few products are purchased without referring to their respective reviews, suggesting that purchasers place equal or even greater value on the recommendations and opinions of other customers compared to seller ratings (Stummeyer and Köber, 2020). 73 % of purchasers prefer qualitative written reviews over star ratings (Fan and Fuel, 2016), and apart from price, reviews are the second most important criterion for purchase decisions (IFH Cologne 2018 in Stummeyer and Köber, 2020). For this we used Amazon rollator reviews as qualitative data for a secondary analysis.

### 2.4 Data collection

We conducted product research on Amazon using the term “rollator” from December 2023 to January 2024. Including criteria where at least forty reviews, only standard all-rounder rollators in the 4-wheeled version (e.g. no outdoor walkers), and only reviews written in English or German. We excluded rollators with several model variants to choose from, as the Amazon rating interface made it impossible or at least difficult to clearly allocate the rating of each model variant. We also excluded discount campaigns and price reductions. To ensure the feasibility of the data collection, we used a stratified random sample in which we formed three purchase price groups (<100 €, 100–200 €, and >200 €). In each of these groups, we collected data from four rollator models. The randomised sample was then selected based on the criteria as follows: We entered the search term “rollator” in the search bar of the German Amazon shop interface and sorted the results by “average customer review”. The reason for this sorting choice is due to the limited display options of the Amazon shop interface. To reduce bias, we decided to sort by high star ratings as an indicator of high quality, to minimise outliers and distortions caused by low-quality products and Amazon-based seller placements. The rollators were then selected based on the three purchase price groups in the order in which they were listed. The individual reviews were than sorted using the filters “most recent”, “verified purchase only”, “all start”, “all formats”, and “text, image, video”. The rationale for including only verified purchases was to reduce the presence of fake reviews in the raw data. Reviews that stood out due to their unusual length or writing style were checked for authenticity using ReviewMeta (2024). Through this process, two reviews were excluded.

### 2.5 Data processing

We manually copied customer reviews into an Excel spreadsheet, recording sequential numbering, star rating, review title, review text, country, year, pictures, and word count. Emojis used in the customer reviews were transcribed based on their meanings (Emojis Wiki, 2024) and counted as one word. We performed no orthographic correction of the reviews. Due to the distortion of the word count by paragraphs, the reviews have been formatted by removing them. To be able to code the raw data efficiently, the Excel spreadsheets were converted into PDF files and uploaded to MAXQDA 2024 (VERBI, 2023). One researcher (MS) carried out data collection and processing. Original German quotes presented in the results section were translated and adapted by a researcher (MS) to preserve their meaning, checked with ChatGPT 4o, and discussed within the research team. Corresponding quotes in the text are marked with *tfG* (translated from German).

### 2.6 Data analysis

For deductive data analysis, we developed a codebook based on the satisfaction dimensions of QUEST 2.0. A code was created for each dimension, with two subcodes – *satisfied* and *dissatisfied* – alongside code descriptions and encoding rules. In the initial phase, anchor examples were identified from the dataset by a researcher (MS). The codebook was then transferred into MAXQDA to facilitate easier coding.

To enhance trustworthiness of the deductive coding process, a partial intercoder agreement test was conducted in MAXQDA with three researchers (MS, AK, LH). Each received a codebook detailing procedure, categories, definitions, anchor samples, and abstraction levels. In the first round, 50 selected reviews (MS) were coded by each researcher and then compared using the partial intercoder agreement test, resulting in a score of *K* = 0.31. Following a reflection within the research team, which included adjustments to the codebook and coding procedure, a second round was conducted with 100 selected reviews (MS), yielding a result of *K* = 0.72 and a third round, also with 100 selected reviews (MS) resulting in *K* = 0.76. This intercoder reliability was accepted by the research team due to the codebook based on QUEST 2.0, in which the segments of the reviews could not always be clearly assigned to just one category. The consolidated codebook was then applied by one researcher (MS) to all 1.028 reviews.

For inductive data analysis, we conducted open coding to identify patterns and themes within the data that were not, or not fully, addressed by the deductive procedure. Segments from Amazon reviews were assigned interim codes in an initial coding step by a researcher (MS), closely adhering to the customers’ wording to avoid limiting interpretative possibilities from the outset. These codes were continuously compared within the dataset and against findings from the narrative literature review (Schmucker et al., 2024). Building on this, frequently occurring codes were identified and consolidated into meta-categories. The conceptual interpretation of thematic relationships, and thereby the connections between meta-categories, were visually represented using the MAXMaps function in MAXQDA.

To enhance trustworthiness of the inductive coding process, the first 25 reviews were independently open coded by three researchers (MS, AK, LH) during a workshop, followed by a joint reflection session of interim coding. In the next step, one researcher (MS) continued open coding across the remaining raw data and developed main categories through focused coding. Resulting interpretations of the main categories were discussed in a tandem (MS, AE) and within the coding group (MS, AK, LH).

### 2.7 Ethics

This study is part of a dissertation project and has been covered by an ethical clearance from the Ethics Committee of the University Hospital of Tübingen. Although we worked exclusively with freely accessible customer reviews, we are ethically committed to ensuring that names and photos of individuals published in the reviews are either not reproduced or anonymised.

## 3 Results

### 3.1 Description of the raw data

The raw data is characterised as mostly short text sections with high information density, distributed over the period from 2015 to 2024, and maintaining an average star rating no lower than 4.34 (see Table 1 ).

**Table 1:** Included rollators by price group, number of reviews, average star rating, and word count.

Rollator	Price group	Number of reviews	Average star rating	Average word count	Time period
JR001100	<100€	100	4,55	21,69	2022/2023
IB101201	<100€	100	4,67	23,04	2019–2023
JR202294	<100€	93	4,62	23,80	2019–2024
TL295395	<100€	101	4,68	24,45	2021–2024
AN396481	100–200€	86	4,53	34,74	2018–2023
AL482582	100–200€	100	4,62	23,13	2022/2023
DM583681	100–200€	99	4,79	27,69	2020–2024
HE9581028	100–200€	71	4,54	23,37	2023/2024
BB682723	>200€	42	4,34	73,71	2021–2023
AC724771	>200€	48	4,53	64,29	2020–2023
DB772856	>200€	85	4,73	40,06	2015–2023
DM857957	>200€	101	4,77	33,01	2019–2023

**Table 2:** Number of deductive codes based on QUEST 2.0 and satisfaction.

Category	Satisfied % (No.)	Dissatisfied % (No.)
Easy to Use	92.4 (452)	7.6 (37)
Comfort	90.0 (412)	10.0 (46)
Effectiveness	84.5 (246)	15.5 (45)
Durability	78.0 (220)	22.0 (62)
Weight	86.3 (208)	13.7 (33)
Service Delivery	83.1 (152)	16.9 (31)
Dimensions	85.5 (153)	14.5 (26)
Safety	73.0 (84)	27.0 (31)
Professional Service	55.1 (49)	44.9 (40)
Adjustments	53.5 (38)	46.5 (33)
Repairs & Servicing	44.0 (11)	56.0 (14)
Follow-Up Service	0.0 (0)	0.0 (0)

### 3.2 Syntheses and interpretation

#### 3.2.1 Deductive

A total of 2423 deductive codes were assigned based on the QUEST 2.0 dimensions, with the categories “Easy to Use” (489 codes) and “Comfort” (458 codes) being the most frequently coded. The category “Follow-up service” could not be coded in the collected data (see Table 2).

The findings demonstrate that “Service Delivery” was by far the most frequently found product service category, due to the nature of written reviews, where users often comment on the delivery experience of their purchased rollators. While most codes were assigned to satisfied categories, this trend shifts in the categories of “Professional Service” and “Adjustments”, where ratings were nearly balanced between satisfied and dissatisfied, with slightly more reviews coded as satisfied. In contrast, the “Repairs & Servicing” category showed a higher proportion of dissatisfied responses.

#### 3.2.2 Inductive

*Expectations for the Rollator.* The expectations for rollators vary according to the user category. We distinguish here between primary, secondary, and tertiary users. Primary users are those who use rollators directly. Secondary users are individuals who occasionally interact with rollators, for instance, by placing the rollator in the trunk of a car or by purchasing and assembling them. Tertiary users are those indirectly impacted, such as prescribers, sales personnel, mechanics, and package delivery drivers. Our analysis yield insights only for primary and secondary users. Expectations focused on product- and product service quality, identified through deductive coding. However, specific aspects remain overlooked, such as expectations of familiar product design *“I already knew this rollator from previous years, which is why I ordered it again. However, for today’s standards, it is too heavy and not easy to manoeuvre*” (No.200, tfG), expectations regarding materials used “*The tires could be rubber, that’s the only drawback*” (No.222, tfG), expectations of product origin “*Since* [anonymous] *is a German company, I assumed the rollators were manufactured in Germany. Upon delivery, I saw ‘Made in China’ on the package*” (No.124, tfG), or expectations related to the user manual “*Good, but manual is not very clear, especially on the fixing of the bottom of the bag*” (No.294). Individual expectations are shaped by personal perceptions of quality, which are often linked to the rollator’s price “*Good quality at a great price*” (No.336, tfG). When the product price is low, users were more likely to accept certain compromises in quality or explain perceived weaknesses in the rollator as a result “*Well, of course, low price doesn’t allow for an overly comfortable weight*” (No.089, tfG). For high-priced models, a certain level of quality was expected in return, “*Thinking for the price it would be a quality walker… I was wrong*” (No.717). Product descriptions from vendors contributed to shaping customer expectations, as users often refer to the product description text in their feedback, “*The rollator is advertised as suitable for both outdoor and indoor use. However, it is only after purchase that the user manual states, ‘The rollator should not be exposed to rain.’ What if I get caught in the rain outside? I believe this information should be included in advertising and not only disclosed after purchase*” (No.510, tfG).

*Responsibility for Procurement.* Besides primary users, secondary users also appear as purchaser and thus as reviewers of rollators. This group comprises various roles within the primary user’s social network. Based on the reviews, we identified partners, children, grandchildren, sons- and daughters-in-law, friends, and other relatives. Secondary users took on the purchasing process due to their caregiving responsibilities for primary users, potentially to compensate for challenges the primary users may face when buying rollators online. Rollators were sometimes purchased as gifts or to encourage potential primary users to start using one, “*After my grandma resisted using a rollator for a long time (her words: ‘Only old people use those,’ my grandma, 89 years young), I bought her this model… After a bit of hesitation, she suddenly can’t go without it*” (No.425, tfG). Purchase decisions were either made jointly between primary and secondary users or as individual choices of secondary users, occasionally reflecting paternalistic behaviour. Besides the desire to support assisted mobility through rollators, secondary users also had personal goals for the purchase, such as wanting to stay mobile together with the primary users, “*I ordered this walking aid for my mother so that I can go out with her anytime. That’s why I specifically looked for a foldable model. Unfortunately, you have to completely unscrew the long bolts every time. Inconvenient! That’s why I deducted points. But it fits perfectly in the trunk of my Fiat 500.*” (No.083, tfG). Besides the personal goal of “…*so that I can go out with her*…” this example also shows how secondary users’ convenience ratings are reflected in Amazon reviews. Whether the purchase by secondary users was successful was primarily evaluated in the reviews using the parameters “uses the rollator / does not use it”, “satisfied / dissatisfied”, and “user experience”. Especially for individual purchasing decisions made by secondary users, reviews sometimes revealed significant pressure to have bought the right rollator, “*This rollator was a perfect hit as a birthday gift. The joy was immense, I did everything right*” (No.365, tfG).

*Delivery and Purchase Process.* In the descriptions of the delivery and purchase process within the reviews, we found comparisons between Amazon and medical supply stores, mostly referring to price, “*It is identical to the standard rollator from the medical supply store. However, the processing and fit are much better. About 100 € cheaper*” (No.108, tfG) and “*The medical supply stores should all look at these prices; the stores are far too expensive. The health insurance companies could save a lot of money*” (No.202, tfG). In the price category >200 €, two reviewers mentioned the possibility of testing the rollator as an advantage, “*…you can try it for 30 days anyway and see if it meets your needs*” (No.756). The delivery process was evaluated positively when the delivery time of the rollators was considered short and when the packaging was perceived as sturdy and safe, “*The rollator was well-packaged and delivered very quickly*” (No.303, tfG). Negative reviews mentioned longer delivery times than promised, poor packaging, missing or broken parts, and incorrect rollator colour. A friendly and efficient customer service was rated positively in such situations. Poor delivery service was mentioned negatively, “*…poor delivery with* [anonymous]*; it was simply left in the building without notification, making it difficult to receive, especially if you need a rollator. A shame*” (No.921, tfG).

*Aesthetic Aspects and Emotions Evoked by the Rollator.* Reviews often addressed the aesthetic aspects of the rollator, which we primarily analysed under the deductive code *Comfort*. Nevertheless, we were able to identify deeper underlying aspects within this category. Models with modern designs and various colourful finishes were perceived positively, which may suggest a previously experienced age-related stigma associated with traditional designs, “*Wow! I was fed up looking like an old lady when using my rollator…*” (No.853). This also suggests that some users do not separate the external appearance of the rollator from their own self-presentation. Like glasses, the rollator as an aid merge into the overall image of how other perceive them, “*Did I mention it is beautiful? I always felt odd in my skinny jeans and modern clothes – using a walker! …I feel confident again. The psychological aspect cannot be overestimated*” (No.756). The use of modern and attractive designs makes it easier for users to use them, “*I’m only 35 so I’m pleased to find something modern*” (No.818), and “*…the design is exceptionally beautiful… it must be a joy to roll around with it*” (No.736, tfG). This highlights that design and appearance of rollators can influence their usage while also evoking positive emotions, “*Such a lovely walker, everyone’s admiring my mother*” (No.878, tfG). The successful use of rollators fosters increased self-confidence and a sense of self-efficacy among users, “*Has given my wife more confidence when out walking*” (No.583), often attributed to an enhanced feeling of safety in mobility, *“I cannot walk unaided and it makes me able to walk safely”* (No.784). This also implies that users may develop a dependency on their rollators, as reflected in concerned reviews that are mistakenly used as a means of contacting the vendor or in statements acknowledging that caring for a relative would not have been possible without it, “*Due to an accident, the front wheel of my rollator has broken. I rely on my rollator, so I would greatly appreciate the quickest possible delivery. I’ll make sure to pay the invoice straight away*” (No.022, tfG) and *“Without this item, I would never have been able to care for my husband at home”* (No.107, tfG). If a rollator fails to meet the trust placed in it, users may experience a significant breach of confidence, often leading to uncertainty about its continued use, “*Last night, the brakes were firmly engaged, yet the rollator still slipped away, and I fell to the floor. I can no longer trust this rollator. Keep your hands off this cheap junk*” (No.031, tfG).

*Impact of Time, Experience, and Wear on Rollator Evaluation.* The influence of time aspects is apparent in the reviews, i.e., in relation to the perceived duration of use. While rollators are predominantly regarded as long-term solutions, especially in the elderly population, two reviews mentioned temporally limited use cases, “*I bought the rollator for my wife to use during the initial period after her hospital stay*” (No.368, tfG), and “*The rollator is excellent. I only need it temporarily”* (No.534, tfG). Progressive illness and age-related physiological changes, which unfold over time, can gradually alter the requirements for rollator usage, making previously adequate solutions insufficient to meet users’ evolving needs, “*I am thrilled with this rollator. Before this, I also had one from them company* [anonymous]*, but that model was too limited and no longer sufficient for my condition*” (No.424, tfG). Further temporal effects emerge both in the personal experiences gained with rollator and in the rollator itself. Users report that an initial adjustment period with a new rollator is quite common, “*The rollator has only been in use for a week and naturally still takes some getting used to*” (No.500, tfG), and that rollators, much like new shoes, often need to be *broken-in* over time, “*…for the first few months I was unable to fold it myself as it was too stiff. It has loosened enough now with repeated use*” (No.773). A form of rollator expertise likely develops over time with continued use, as one reviewer described a model as too cumbersome to push for experienced users but considered this characteristic an advantage for beginners, “*For beginners with rollators, it’s probably a good thing that it’s a bit cumbersome. This way, they can gradually get familiar with how it handles*” (No.387, tfG). This expertise may diminish following a rollator model change, requiring users to adapt to new handling, “*In terms of the handling, I now have to adjust a bit since the front wheels don’t stay fixed like they did on my previous rollator*” (No.884, tfG). In addition to the *broken-in* effect described above, rollators also change over time due to wear and tear. Assessments of quality and satisfaction with durability can be time-sensitive and may evolve both positively, i.e., due to the pleasant surprise of a rollator’s durability, and negatively over time, “*Can I send the rollator to you for repair? The tires are already worn out and the brakes are not working properly. Please send me a return email for repair. For your information, the rollator is only about 4 to 5 months old*” (No.068, tfG). Regardless of whether the use of the rollator can be classified as heavy use or not, “[The rollator] *It has had a very hard life of daily transporting shopping, laundry, picnic stuff and craft materials. It has also been on holidays, across beaches and fields*” (No.772), rollators require regular maintenance of wear-and-tear components and movable parts, “*The rear right wheel is secured with a nut that has a standard right-hand thread. In my case, this led to the nut gradually loosening over approximately one year. It is possible that the nut (a self-locking version) was of subpar quality. Therefore, it is essential to regularly check that nuts are securely fastened*” (No.077, tfG).

## 4 Discussion

### 4.1 Rollator from the perspective of Amazon reviewers

The deductive coding results showed that *Ease of Use, Comfort*, and *Effectiveness* were the most frequently assigned categories. For *Ease of Use*, we found similar results to those in our literature review (Schmucker et al., 2024), where it ranked first, and in rollator user surveys conducted with the QUEST 2.0 by Samuelsson and Wressle (2008), where *Ease of Use* ranked first, and by Wressle and Samuelsson (2004), where it ranked second. *Comfort* appears on second rank in our review (Schmucker et al., 2024) and in Wressle and Samuelsson’s study (2004), ranking third. In contrast, *Effectiveness*, which ranks fourth in the review (Schmucker et al., 2024), does not appear among the top three dimensions in any of the mentioned surveys. Whereas *Safety* was ranked third in our review (Schmucker et al., 2024), second In Samuelsson and Wressle (2008), and first in Wressle and Samuelsson (2004), in this analysis, *Safety* did not appear in the top three. These comparisons are interesting when considering the content of Amazon reviews as reflective of the product aspects most important to customers. A possible explanation lies in the varying user groups. While rollator surveys included only primary users (Samuelsson and Wressle, 2008; Wressle and Samuelsson, 2004), Amazon reviews also reflect the perspectives of secondary users, such as informal caregivers i.e. partners, children, and other relatives. The frequent occurrence of *Service Delivery* codes, compared to the literature review results (Schmucker et al., 2024), and the complete missing of *Follow-Up Service* codes can be attributed to the nature of our data source. All other Service Dimensions of QUEST 2.0, particularly shipping related topics, occur relatively frequently, even though this is prohibited by Amazon’s Guidelines for Customer Reviews and Ratings (Amazon, 2024).

It is obvious that relatives often purchase rollators out of care. However, less explored is the purchase of rollators not initiated by primary users themselves, but by relatives who believe these devices can improve their loved ones’ quality of life. These purchases may at times reflect additional personal goals, such as enabling shared mobility with the affected individual, ensuring the rollator fits into their own car, or facilitating home care that would otherwise be challenging without. This highlights the importance of including secondary users in the requirements assessment for new rollator developments. The deductive and inductive findings highlight the importance of service quality in rollator use, reinforcing Boerema et al.’s call to shift the current focus of companies from rollator design, which mainly encompasses material aspects, to product-service systems. This refers to incorporating non-materialistic needs into product design, such as the provision of knowledge, to create additional value and thereby achieve higher quality, e.g., integrated systems that assist with selecting and booking a taxi after a visit to a general practitioner (2017). In our view, before integrating additional services, it is crucial to address more fundamental aspects to ensure the basic functions of rollators are utilised safely. This requires providing knowledge on when a rollator needs maintenance, if it is properly adjusted, and if it is being used correctly. In addition to technical solutions such as gait and posture analysis (Mandel et al., 2020) or indicators for wear parts, such as brakes, to signal when they should be replaced, companies should provide easy accessible information about their products. Rollator design should also ensure that adjustments, as well as the replacement of wear parts, can be carried out easily and preferably without the needs for tools. Such product features could exceed users’ quality expectations, which, in addition to ensuring higher quality and safer application, could foster greater customer loyalty and stronger brand identification.

Viewing the rollator as a technical actor in managing everyday life from a non-dualistic perspective of Actor-Network Theory (Latour, 2019), several influences can be derived from the interpreted results. In addition to personal factors such as health and physical condition, the integration into social support networks, such as family and friendships, as well as financial resources, likely have an impact on the successful use of a rollator. The individual’s positive or rather negative attitude toward the rollator, often rooted in anticipated age-related stigma, is frequently addressed by manufacturers through modern design. This highlights that rollator, for some users, are more than mere assistive devices; they become part of their appearance, much like a stylish pair of eyeglasses. In this regard, and in statements describing a high dependency on rollators, there are indications of possibly temporary integrations of human and assistive device, forming a human-machine hybrid (Latour, 2019) in certain usage situations, such as situations like dining at an upscale restaurant with a worn, scratched rollator, or being unable to continue shopping due to a broken wheel. In addition to widely recognised actors, such as context (outdoor/indoor) and the resulting usage differences (weather conditions, spatial factors, varying surfaces, barriers, etc.), temporal aspects exhibit agency in the form of experience and in the form of material wear. Experience in handling a specific rollator facilitates its use and, according to some users, allows for the usage of more advanced, potentially less stable but more mobile rollators. However, any change in model requires an adjustment period for both the user and the device. The wear and tear of rollator components, such as brakes and tires, depends not only on the frequency of use but also on the usage environment, the user’s experience, and their (or their social networks) maintenance skills. For instance, inexperienced and cautious users often tend to operate the rollators with the brakes slightly engaged, leading to significantly faster brake wear and reduced efficiency. Experience is also reflected in the recognition of rollator features that, while nearly identical across most rollators, are not immediately apparent without prior knowledge. In the Amazon reviews, we found instances of confusion and frustration regarding the perceived absence of parking brakes on rollators. However, one customer clarified, stating, “*Only the assembly and manual instructions need improvement. I only discovered by chance how the parking brake works – push the brake lever down!*” (No.570, tfG). According to Norman’s (2013) concept of the affordance of objects, there is a lack of clear signifiers indicating how to use the parking brake. Most users cannot anticipate this function from their experience with other devices and transfer it to the rollator. All of this supports Janda’s (2019) statement that usability is not a static characteristic of technology and thus not merely a product attribute. Instead, it is a qualitative characteristic about the technology usage, which is continuously shaped by both materialistic and non-materialistic actors. This highlights the need for usage support in the form of training, rollator maintenance and adjustment, as well as individualised technology assessments to respond to changing needs over time.

### 4.2 Rollators from a nursing science perspective

Our results show how important rollators are for maintaining the mobility of those affected and how they affect their ability to carry out Activities of Daily Living (Roper et al., 2000) and thus remain in their own home. Compared to the highly complex technologies much discussed in nursing science such as Ambient Assisted Living, AI, and robotics, rollators are a relatively simple device that nevertheless offers the possibility of restoring autonomy and self-care in the face of increasing need for care due to mobility restrictions. The analysed Amazon reviews illustrate the influence of technical actors – in this case, the rollator – in shaping care situations. Accordingly, certain caregiving tasks are only possible with the integration of technical devices (Manz, 2015), which in some situations leads to a strong dependence on assistive technology. For this reason, nursing science should also consider the practical impact of assistive devices such as rollators in daily care settings. This is where nurses should take a role in providing advice and training, both at the point of purchase and during use. In Germany, advice and fitting is mostly only provided when a rollator is purchased from a medical supply store (Lindemann and Freiberger, 2022), while this support is often lacking when a purchase is made via online shops, retail stores, and second hand. Nurses should know which rollators for different usage contexts and their individual adaptations to the users. Community nurses could be particularly effective in this regard, as they are often closely familiar with the daily care routines and living environments of care-dependent individuals and their relatives. This proximity enables them to identify necessary adjustments and maintenance requirements for rollators at an early stage. In addition, nurses can instruct and train relatives on which repairs and adaptations can be carried out themselves and when a professional service should be called in or when a change of aid or the addition of further aids becomes necessary due to changes in condition.

### 4.3 Benefits of analysing Amazon reviews

Amazon product reviews have proven to be an interesting data source for understanding user requirements for rollators in our study. One notable advantage is the accessibility to user opinions, which is often challenging to achieve through traditional user surveys, particularly for assistive devices. Amazon reviews are particularly interesting because they offer user-generated insights about rollators that are not influenced by specific scientific research agendas. This may make them less prone to selective bias often associated with surveys designed to predefined research questions. However, Amazon product reviews carry other biases due to their context of origin. Request to write reviews are sent by Amazon via email a few weeks after receiving the rollator. In this short timeframe, users may not yet be able to evaluate all product aspects, such as durability. This is reflected in statements like, “*To give an accurate assessment, I need to use the rollator extensively; a few days are not enough for an evaluation*” (No.990, tfG). The review of issues such as ordering, shipping, packaging, product condition and damage, shipping costs, and delivery speeds violates Amazon’s Community Guidelines. This restriction is justified with the statement, “*Why not? Community content is meant to help customers learn about the product itself, not someone’s individual experience ordering it*” (Amazon, 2024). Although it is possible to rate sellers and shipping separately elsewhere in the Amazon platform, this separation of rollator characteristics from delivery contradicts the product-service-system understanding. It also disregards the fact that these service aspects influence the overall evaluation of the rollator usability. Additional violations of Amazon’s guidelines found in the study included comments on prices and availability, often noting that the products were cheaper on Amazon compared to medical supply stores.

## 5 Limitations

This study has several limitations that should be acknowledged. Due to a lack of recent studies on rollators use, older data had to be used to justify and contextualise the present research. Within the analysed reviews, it was not always possible to clearly distinguish between primary and secondary users. In addition, the nature of online reviews introduces inherent biases: the influence of Amazon’s request to write product reviews, the associated guidelines, and self-selections bias, whereby users with particularly positive or negative experiences are more inclined to leave a review, may have shaped the content. Moreover, the dataset is limited to German and English reviews, with a higher German proportion. Since we accessed Amazon using a German IP address, it remains unclear to what extent this may have influenced the selection and ranking of displayed reviews.

## 6 Conclusions

The results of this study show the complexity of the usability of rollators and their dependence on various materialistic and non-materialistic factors. By analysing Amazon rollator reviews, we demonstrated that these reviews can be a valuable source for understanding user requirements, including perspectives not traditionally captured, such as those of secondary users (e.g., family members and caregivers). Our findings also highlight the challenges of interpreting usability as a static product attribute. Instead, usability emerges dynamically from the interaction of users with the device within the spatial and social context. This broadens the understanding of rollator usability and emphasises the need to understand rollators as product-service systems. Nurses should play a stronger role in the training and advising on rollators, as they are often closer to the everyday lives and living environments of users and their relatives, enabling them to initiate necessary adjustments that often only become apparent over time. In addition to assessing the context of use, i.e. to prevent falls caused by trip hazards, the nursing perspective should also extend to aspects of the rollator’s condition, such as the functionality of the brakes, to facilitate necessary maintenance work in medical supply stores. In clinical settings, nurses should assess the home environment and individual expectations regarding rollator use before prescribing them to recommend specific rollator models or home adaptations. Manufactures should focus on intuitive, easy-to-maintain product design, while vendors should provide ongoing support, e.g. in the form of training and maintenance resources. Nursing science could play a crucial role in incorporating the user perspective into the development of rollators and exploring the necessary aspects of ongoing support, making an important contribution to improving rollator provision. Finally, this study highlights the potential of non-traditional data sources like online reviews in assistive devices research, although additional research is needed to better understand the opportunities and limitations of this data source.

## Funding

The German Federal Ministry of Education and Research (BMBF) supported this work under funding code [No. 03FHP115].

## CRediT authorship contribution statement

**Marcel Schmucker:** Methodology, Project administration, Conceptualization, Writing – original draft, Formal analysis. **Andreas Küpper:** Formal analysis, Validation, Writing – review & editing. **Laura Hahn:** Formal analysis, Validation, Writing – review & editing. **Cornelia Mahler:** Supervision, Writing – review & editing. **Astrid Elsbernd:** Supervision, Writing – review & editing, Methodology.

## Declaration of competing interest

The authors declare the following financial interests/personal relationships which may be considered as potential competing interests:

Marcel Schmucker reports financial support was provided by Esslingen University of Applied Sciences - Campus Flandernstrasse. If there are other authors, they declare that they have no known competing financial interests or personal relationships that could have appeared to influence the work reported in this paper.

## Data Availability

The raw data used in this study consists of Amazon reviews and is available as supplementary material. Due to ethical and privacy considerations, any personally identifiable information, including company names and product models, has been removed. Further inquiries regarding the dataset can be directed to the corresponding author.

## Appendix Supplementary materials

Image, application 1

Image, application 2

Image, application 3
